# Validating Predictions from Climate Envelope Models

**DOI:** 10.1371/journal.pone.0063600

**Published:** 2013-05-23

**Authors:** James I. Watling, David N. Bucklin, Carolina Speroterra, Laura A. Brandt, Frank J. Mazzotti, Stephanie S. Romañach

**Affiliations:** 1 Ft Lauderdale Research and Education Center, University of Florida, Ft Lauderdale, Florida, United States of America; 2 U.S. Fish and Wildlife Service, Ft Lauderdale, Florida, United States of America; 3 Southeast Ecological Science Center, U.S. Geological Survey, Ft Lauderdale, Florida, United States of America; University of Bern, Switzerland

## Abstract

Climate envelope models are a potentially important conservation tool, but their ability to accurately forecast species’ distributional shifts using independent survey data has not been fully evaluated. We created climate envelope models for 12 species of North American breeding birds previously shown to have experienced poleward range shifts. For each species, we evaluated three different approaches to climate envelope modeling that differed in the way they treated climate-induced range expansion and contraction, using random forests and maximum entropy modeling algorithms. All models were calibrated using occurrence data from 1967–1971 (*t_1_*) and evaluated using occurrence data from 1998–2002 (*t_2_*). Model sensitivity (the ability to correctly classify species presences) was greater using the maximum entropy algorithm than the random forest algorithm. Although sensitivity did not differ significantly among approaches, for many species, sensitivity was maximized using a hybrid approach that assumed range expansion, but not contraction, in *t_2_*. Species for which the hybrid approach resulted in the greatest improvement in sensitivity have been reported from more land cover types than species for which there was little difference in sensitivity between hybrid and dynamic approaches, suggesting that habitat generalists may be buffered somewhat against climate-induced range contractions. Specificity (the ability to correctly classify species absences) was maximized using the random forest algorithm and was lowest using the hybrid approach. Overall, our results suggest cautious optimism for the use of climate envelope models to forecast range shifts, but also underscore the importance of considering non-climate drivers of species range limits. The use of alternative climate envelope models that make different assumptions about range expansion and contraction is a new and potentially useful way to help inform our understanding of climate change effects on species.

## Introduction

Climate change is one of the major conservation issues of the twenty-first century. Because the effects of increasing greenhouse gas are expected to exacerbate climate change over the course of the twenty-first century and beyond [1], models are an important tool for anticipating potential future effects of climate change and identifying proactive mitigation and adaptation strategies. Climate envelope models (CEMs) establish species-climate relationships that can be extrapolated in space and time [2]. Because climate is one of the major filters determining broad patterns of species distribution [3], [4] and because models can be constructed using relatively simple statistical models and data inputs [2], CEMs have become a widely-used tool for forecasting climate change effects on species distributions [5], [6]. However, CEMs have been criticized as lacking a sound theoretical foundation, making unrealistic assumptions about species-climate relationships (e.g., assuming niche conservatism [7]), and too-easily leading to unjustified conclusions [3], [4], [7]. In some cases, empirical data refute the importance of climate change in underlying contemporary range shifts, even for species presumed to be vulnerable to climate change [8]. Here, we use independent data on changes in the distribution of selected breeding birds in North America from 1967–71 to 1998–2002 to evaluate the ability of three alternative models, including one with no climate change, to correctly classify species and absence.

If CEMs are to be used as a robust natural resource management tool, their ability to accurately forecast species’ distributional shifts [9] or population trends [10] needs to be evaluated with field data. Relatively few studies that have evaluated CEMs by calibrating models with historical data (i.e., an initial time period, *t_1_*) and evaluating them with data from a future time period for which there are empirical data on climate and species occurrence (*t_2_*). Those studies that have been conducted have differed in their assessments of CEM performance, with one study suggesting that CEMs are capable of making predictions that are of fair to good performance [9], one indicating relatively poor predictive performance [11], and another showing mixed results [8]. To some degree, the determination of a model’s ability to accurately forecast a species’ future distribution depends on the metric used to evaluate model performance [12], which depends in part on the relative importance of omission and commission errors [13]. Although some have suggested that when projecting future climate change effects, omission errors (i.e., failing to predict a known occurrence) are more serious than commission errors (predicting species presence in areas where it is not known to occur; [13], [14]), the decision of how to balance omission versus commission errors is highly case-specific.

**Figure 1:**
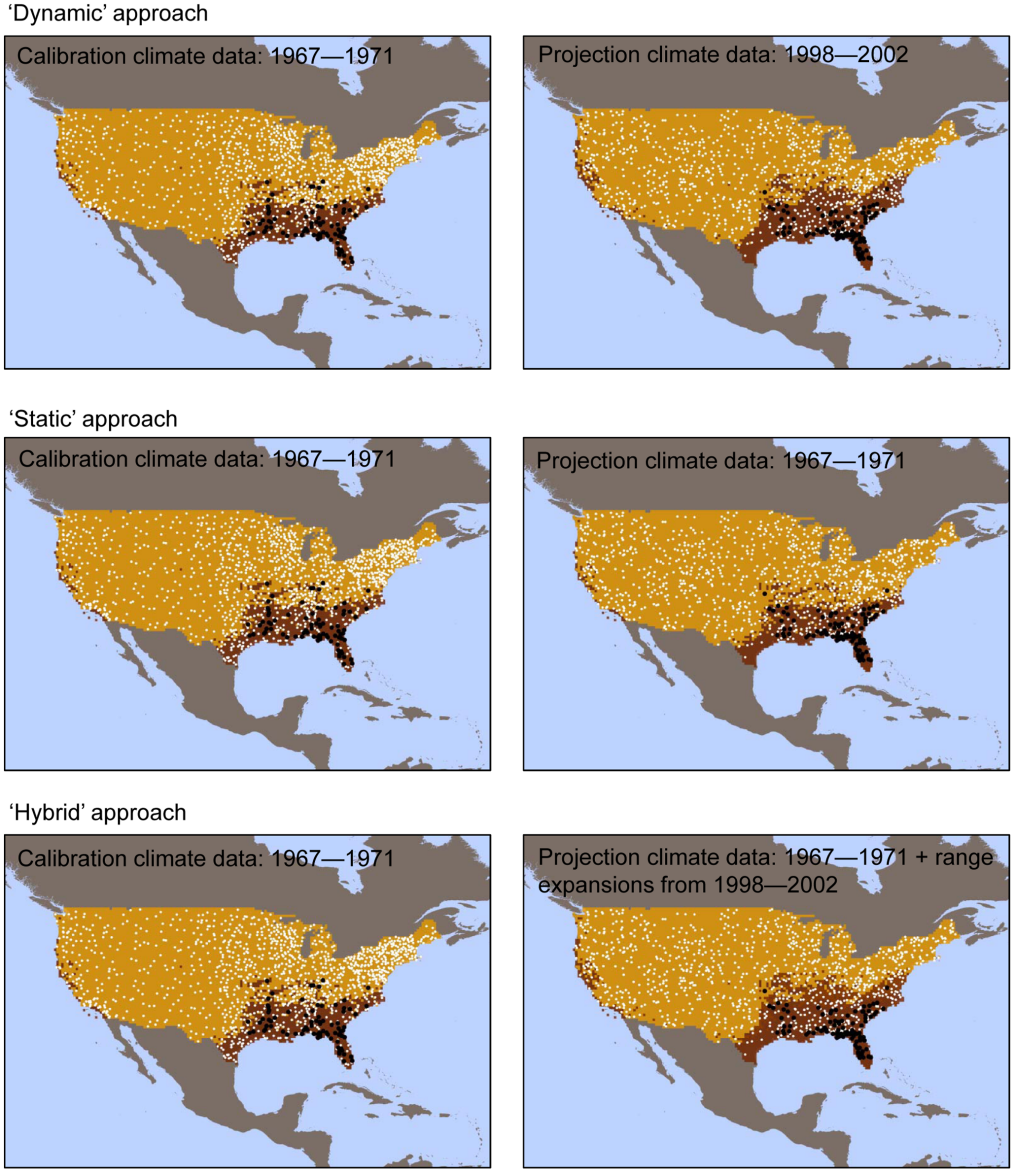
Examples of the three approaches to construction and validation of climate envelope models. All models are calibrated with occurrence data and climate conditions from 1967–1971 (left-hand panels), and validated with occurrence data from 1998–2002 (right hand panels). Black circles indicate species presence and white dotes indicate species absence. The prediction map against which occurrences are validated using the dynamic approach represent the climate envelope under 1998–2002 conditions and for the static approach the prediction map represents the 1967–1971 climate envelope. Under the hybrid approach, areas of range expansion between 1967–1971 and 1998–2002 are merged with the 1967–1971 climate envelope to create a third prediction map.

**Figure 2:**
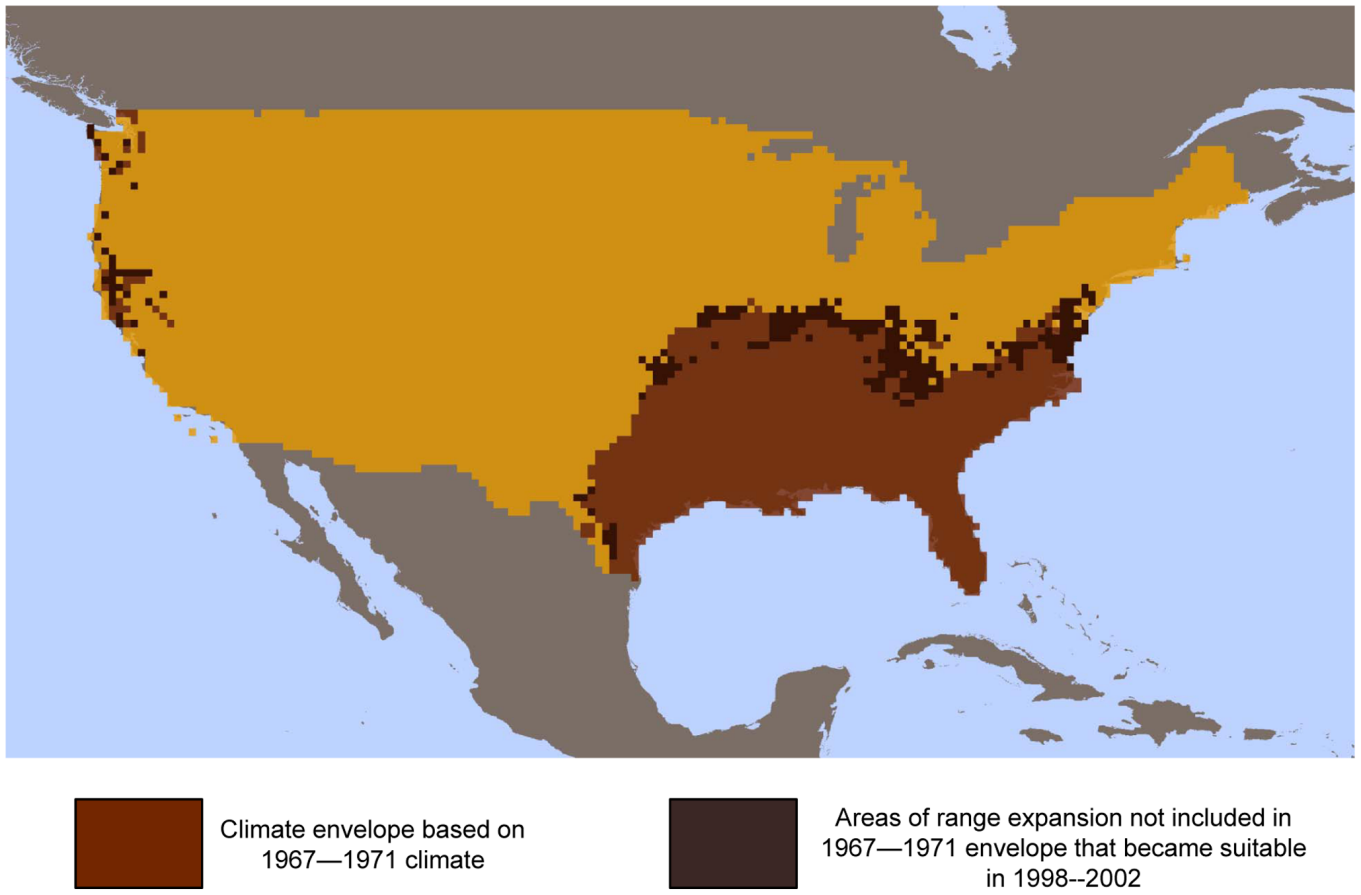
Example map illustrating a ‘hybrid’ approach to climate envelope model construction. Areas indicated in black are included in the 1998–2002 projection, but were not part of the initial climate envelope. These areas range expansion between 1967–1971 and 1998–2002 are merged with the initial 1967–1971 climate envelope to create a hybrid prediction map.

To determine the ability of CEMs to forecast geographic range shifts presumed to have occurred in response to recent climate change, we evaluated performance of CEMs using metrics describing both omission and commission error. We compared three alternative approaches to model construction and evaluation (Figure 1) that differed in the way they described areas of expansion and contraction of the climate envelope. The first approach incorporated climate change between *t_1_* and *t_2_* by calibrating a model with the *t_1_* occurrences and *t_1_* climate data, extrapolating the model into *t_2_* climate conditions, and evaluating model classification with the *t_2_* occurrence data. We refer to this as the ‘dynamic’ approach to climate envelope modeling. Under a dynamic model, the climate envelope was allowed to both contract and expand in response to changing climate. The second approach calibrated a model with the *t_1_* occurrence and *t_1_* climate data and evaluated the ability of that model to correctly classify *t_2_* occurrences. In other words, the second ‘static’ approach tested the ability of a model that described no change in climate suitability (e.g., neither expansion nor contraction of the climate envelope) to classify the *t_2_* occurrences. A third ‘hybrid’ approach calibrated a model with the *t_1_* occurrences and *t_1_* climate data and projected the model into *t_2_* climate conditions. We then identified those portions of the map that changed from being outside of the climate envelope in *t_1_* to within the climate envelope in *t_2_* (i.e., the areas in which the climate envelope expanded between the two time steps, Figure 2), appended those areas of expansion to the *t_1_* climate envelope, and evaluated the ability of the model to correctly classify the *t_2_* occurrences. This approach explicitly assumes that areas of climate suitability at *t_1_* will remain suitable at *t_2_*, while also considering newly suitable areas when classifying *t_2_* occurrences. We did not eliminate areas where the climate envelope contracted between 1967–71 and 1998–2002 because recent work suggests the potential for long-term persistence of sink populations experiencing negative growth rates [15].

## Materials and Methods

Species occurrences for model calibration and evaluation were drawn from the Breeding Bird Survey (BBS) dataset [16]. Breeding Bird Survey data are collected annually by thousands of volunteers who record species observations along fixed survey routes, and are a key source of long-term population data for North American breeding birds. To define the pool of species for which models would be constructed, we searched the primary literature for studies of latitudinal range shifts in birds; three studies presented data for multiple species and were used to create our species pool ([17], [18], [19]). We created models for species known on the basis of previously published data to have experienced a poleward distributional shift (either north or south). Hitch & Leberg [17] tested for significant distributional shifts among species included in their study so we included species for which their tests were significant at α ≤ 0.05. In the remaining two studies, the significance of range shifts was not tested for individual species, and different metrics were used to describe range shifts. In lieu of a significance test, we developed operational criteria for including species in our study. For species reported on in La Sorte & Thompson [18] we included species for which the slope of the relationship describing movement of the northern range boundary through time was 5< slope<−5 (e.g., the species that had experienced the most dramatic range shifts in their study). For species reported on in Zuckerberg et al. [19], we included species for which the northern or southern range boundary shifted >50 km. In all cases, migratory species were excluded from consideration because fine temporal resolution climate data for evaluating models are not available for much of the Neotropics. Modeled species therefore had to be resident in and restricted to the contiguous United States (two species, the Wild turkey *Meleagris gallopavo* and Gambel’s quail, *Callipepla gambelii* also occur in parts of Mexico, but because most of their range is within the contiguous United States, they were included in our analysis) and have experienced a significant poleward distributional shift according to criteria described above. Across the three studies, our selection criteria identified 12 species for modeling (Table 1).

**Table 1:** Summary statistics for climate envelope models for twelve species of resident North American breeding birds.

Species	CommonName	No. presences1967–1971/1998–2002	Rangeshift	Thresholdcriterion	AUC (static)	AUC (dynamic)	True skill statistic	Sensitivity(dynamic approach)	Specificity(dynamic approach)	Sensitivity(static approach)	Specificity(static approach)	Sensitivity(hybrid approach)	Specificity (hybrid approach)
**Phasianidae**													
*Meleagris gallopavo*	Wild turkey	73/967	North	Prevalence									
RF					0.577±0.030	0.613±0.032	0.159±0.051	0.425±0.043	0.734±0.036	0.295±0.028	0.844±0.033	0.591±0.041	0.610±0.044
Maxent					0.650±0.029	0.652±0.032	0.091±0.047	0.957±0.022	0.134±0.056	0.904±0.034	0.204±0.051	0.995±0.007	0.031±0.020
*Centrocercus urophasianus*	Greater sage grouse	20/60	South	Prevalence									
RF					0.943±0.020	0.927±0.021	0.721±0.060	0.951±0.055	0.768±0.033	0.955±0.054	0.794±0.033	0.997±0.016	0.591±0.041
Maxent					0.951±0.016	0.944±0.015	0.652±0.047	0.999±0.007	0.653±0.048	0.993±0.020	0.610±0.058	0.999±0.001	0.398±0.067
*Tympanuchus cupido*	Greater prairie chicken	19/35	North	Prevalence									
RF					0.827±0.077	0.838±0.067	0.479±0.158	0.749±0.153	0.745±0.038	0.751±0.140	0.779±0.030	0.934±0.093	0.577±0.042
Maxent					0.875±0.041	0.910±0.032	0.095±0.091	0.999±0.001	0.095±0.091	0.999±0.001	0.082±0.071	0.999±0.001	0.013±0.021
*Callipepla gambelii*	Gambel’s quail	27/78	North	Prevalence									
RF					0.943±0.021	0.938±0.021	0.723±0.058	0.953±0.051	0.768±0.033	0.919±0.059	0.836±0.026	0.996±0.013	0.625±0.043
Maxent					0.955±0.015	0.948±0.013	0.669±0.034	0.999±0.001	0.670±0.034	0.997±0.018	0.710±0.038	0.999±0.001	0.463±0.039
**Picidae**													
*Melanerpes erythrocephalus*	Red-headed woodpecker	624/852	South	Maximum kappa									
RF					0.834±0.015	0.814±0.018	0.402±0.041	0.929±0.017	0.472±0.038	0.910±0.016	0.581±0.030	0.994±0.005	0.215±0.029
Maxent					0.877±0.016	0.870±0.016	0.613±0.035	0.905±0.019	0.709±0.032	0.877±0.021	0.743±0.029	0.987±0.007	0.420±0.031
*Melanerpes carolinus*	Red-bellied woodpecker	599/830	North	Maximum kappa									
RF					0.776±0.020	0.766±0.019	0.311±0.037	0.912±0.017	0.398±0.033	0.754±0.028	0.579±0.031	0.977±0.009	0.201±0.026
Maxent					0.852±0.017	0.806±0.019	0.466±0.036	0.852±0.022	0.614±0.029	0.748±0.030	0.768±0.026	0.962±0.012	0.397±0.031
**Corvidae**													
*Corvus ossifragus*	Fish crow	153/404	North	Prevalence									
RF					0.933±0.014	0.912±0.015	0.605±0.051	0.755±0.050	0.851±0.022	0.736±0.044	0.931±0.016	0.938±0.025	0.741±0.026
Maxent					0.938±0.012	0.921±0.013	0.702±0.034	0.919±0.027	0.783±0.026	0.879±0.031	0.849±0.021	0.989±0.011	0.605±0.033
**Paridae**													
*Poecile carolinensis*	Carolina chickadee	468/791	North	Maximum kappa									
RF					0.911±0.013	0.914±0.011	0.686±0.030	0.956±0.016	0.730±0.024	0.474±0.034	0.974±0.009	0.980±0.011	0.662±0.029
Maxent					0.935±0.011	0.877±0.015	0.674±0.029	0.957±0.015	0.716±0.027	0.499±0.043	0.966±0.011	0.978±0.011	0.653±0.030
**Troglodytidae**													
*Thryothorus ludovicianus*	Carolina wren	535/689	North	Maximum kappa									
RF					0.815±0.018	0.868±0.014	0.451±0.030	0.991±0.007	0.459±0.030	0.828±0.023	0.602±0.025	0.999±0.002	0.248±0.022
Maxent					0.861±0.017	0.875±0.018	0.662±0.032	0.960±0.017	0.702±0.028	0.847±0.023	0.766±0.027	0.994±0.006	0.454±0.032
*Thryomanes bewickii*	Bewick’s wren	246/440	South	Prevalence									
RF					0.780±0.024	0.725±0.023	0.332±0.041	0.824±0.030	0.507±0.031	0.783±0.034	0.658±0.032	0.961±0.020	0.307±0.032
Maxent					0.774±0.024	0.717±0.024	0.335±0.044	0.881±0.028	0.454±0.041	0.829±0.037	0.611±0.033	0.982±0.012	0.249±0.033
**Emberizidae**													
*Aimophila aestivalis*	Bachman’s sparrow	81/95	South	Prevalence									
RF					0.962±0.012	0.954±0.015	0.762±0.041	0.976±0.035	0.785±0.026	0.955±0.042	0.859±0.021	0.999±0.004	0.657±0.031
Maxent					0.965±0.010	0.957±0.013	0.716±0.031	0.999±0.004	0.716±0.031	0.986±0.021	0.772±0.028	0.999±0.001	0.536±0.037
**Icteridae**													
*Agelaius tricolor*	Tricolored blackbird	15/29	North	Prevalence									
RF					0.942±0.044	0.901±0.054	0.654±0.127	0.866±0.113	0.786±0.043	0.919±0.098	0.833±0.035	0.984±0.049	0.659±0.052
Maxent					0.954±0.019	0.940±0.030	0.670±0.065	0.976±0.079	0.694±0.069	0.983±0.065	0.727±0.064	0.997±0.020	0.515±0.121
Average ±1 SD	RF												
	Maxent												

Three approaches to climate envelope model evaluation were compared, a dynamic approach in which a model calibrated on conditions at *t_1_* (here 1967–1971) was projected to predict occurrences at *t_2_* (here 1998–2002), a static approach in which *t_2_* occurrences are predicted using the *t_1_* climate data, and a hybrid approach in which *t_2_* occurrences are predicted using a model that allows for range expansion, but not range contraction at *t_2_*.

Following Hitch & Leberg [17], we used two five-year time periods for model development, the first for calibration and the second for evaluation. Models were calibrated using *t_1_* observations made on BBS routes (e.g., between the years 1967–1971). Repeat observations of a species from a survey route were removed such that if a species was observed at any point during the five year *t_1_* period, it was counted as a single presence. Latitude and longitude coordinate data for routes were obtained from the BBS database ([16]; coordinates are expressed as a single latitude and longitude observation for each 24.5 mile route). Using the coordinate data for each survey route, we extracted the values of seven climate variables at routes known to be occupied by each species. We also selected 1000 random routes from which focal species were unobserved and assumed to be absent, and extracted the values of the same seven variables. Climate variables included were: annual precipitation, precipitation of the driest month, precipitation of the wettest month, mean annual temperature, temperature annual range, maximum mean monthly temperature and minimum mean monthly temperature. Models were evaluated with occurrence data from *t_2_* (1998–2002), which were compiled from BBS survey routes as described for the *t_1_* data. Climate data were obtained from the PRISM dataset (PRISM Climate Group, Oregon State University, August 2011, and average values for all variables were calculated for each of the two five year periods used in our study. Because the PRISM data were downloaded at a resolution of 4×4 km, we resampled the climate grids to 40 km×40 km to approximate the resolution of the BBS survey routes (24.5 miles = ∼39.4 km).

Models were constructed using two different algorithms: the random forest algorithm, which classifies observations (e.g., species presence/absence) based on an iterative, recursive partitioning of observations into the most homogeneous subsets possible [20], and maximum entropy, which calculates a species’ probability of occurrence based on knowledge of environmental conditions at sites known to be occupied by the species and background environmental conditions [21], [22]. Here, we used the 1000 random absences for each species to calculate the environmental background for maximum entropy modeling. Random forest models and all other statistical analyses were conducted in R [23] and maximum entropy modeling was done using the MaxEnt software package [21], [22] using default settings.

We evaluated performance of all CEMs by constructing models with calibration data (*t_1_*) and testing them with *t_2_* occurrences. Four criteria were used to assess model performance: the area under the receiver-operator curve (AUC), the true skill statistic, sensitivity and specificity [12]. The AUC metric ranges from 0–1 and measures the tendency for a random presence point to have a higher predicted probability of climate suitability than a random background point. In addition to using AUC to evaluate the extrapolated model (based on *t_2_* climate and occurrences), we also calculated AUC using a static model in which the *t_2_* occurrences were evaluated against *t_1_* climate conditions. We expected that species whose ranges were shifting in response to climate change would have greater AUC values using the *t_2_* climate data compared with the static AUC calculation. Like AUC, the true skill statistic also ranges from 0–1, but is independent of species prevalence [24]. Sensitivity measures the proportion of correctly classified presences in the test dataset, whereas specificity measures the proportion of correctly classified absences; both metrics range from 0–1. Sensitivity is a measure of omission error (high sensitivity = low omission), and specificity is a measure of commission error (high specificity = low commission). A number of authors have suggested that the ‘best’ models should achieve low rates of omission (i.e., they should accurately classify presences) even if commission error is relatively high, because at least some commission error is not truly error but rather reflects our incomplete knowledge of species distributions or the identification of environmentally suitable area that is inaccessible to species because of dispersal barriers, species interactions or other factors [13], [14]).

Because two of our performance metrics describe the ability of a model to correctly classify presences and absences, they require the user define the threshold probabilities at which presence is differentiated from absence. We used two alternative criteria to determine that threshold. One criterion converted continuous probabilities into a categorical prediction by identifying the threshold that maximized Cohen’s kappa, a model performance metric that measures overall classification ability [25]. To identify this threshold, we ran five replicate model runs using random subsets of the species occurrence data in the calibration dataset (1967–1971) for each 0.01 unit change in threshold between.01 and 0.99 and calculated kappa for each randomization (using a 75–25% training-testing partition of the occurrence data). We calculated the average kappa for each incremental change in the threshold to identify the threshold at which kappa was maximized. A second criterion used a prevalence-based approach to defining the threshold used to calculate kappa [25]. We calculated the prevalence of each species as number of occurrences/(number of occurrences +1000) because all CEMs used 1000 absence points, and used the estimate of prevalence as the threshold for converting probability into categorical predictions. We calculated each threshold (maximum kappa and prevalence) once for each species, and report the threshold that resulted in the greatest model sensitivity (e.g., best classified species presences at *t_2_*). We calculated all performance metrics using a 75–25% training-testing split on 100 random partitions of the occurrence data, and tested for significant effects of algorithm and approach on AUC, the true skill statistic, sensitivity and specificity using generalized linear mixed-effects models [26] with a binomial distribution and a logit link. Algorithm and approach were tested as fixed effects, and species were treated as a random effect. The significance of fixed effects and their interaction was tested as the likelihood ratio between the full model and a model with the effect being tested removed.

The dynamic, static and hybrid approaches to model evaluation differ in the extent to which they treat range expansion and contraction (see above). To understand how model performance varied as a function of classification specifically in areas of range change, we calculated the proportion of *t_2_* presences and absences of each species that occurred in areas of range expansion or contraction between 1967–71 and 1998–2002 using the dynamic approach to model evaluation. We expected that the hybrid approach would result in increased sensitivity compared with the dynamic approach because the hybrid approach assumes no range contraction and therefore maximizes the area of predicted suitability. We further expected that the hybrid approach would improve sensitivity the most for those species for which the dynamic approach resulted in the greatest number of misclassified presences. In other words, models assuming no range contraction should yield the biggest gains in sensitivity for species that continue to persist in areas where range contraction is predicted under the dynamic approach. Therefore, we used linear regression to determine whether species-by-species differences in sensitivity between the dynamic and hybrid approaches were associated with proportions of misclassified presences (i.e., those occurring in areas of range contraction) using the dynamic approach. We also wanted to determine whether species-specific gains in model sensitivity under a hybrid approach were related to species traits. We reasoned that if the gain in sensitivity achieved using the hybrid approach is indeed greatest for species that maintain populations in areas deemed unsuitable by a dynamic CEM, there may be a positive effect of niche breadth on such resistance, much as has been described for the relatively generalist species that persist in fragmented landscapes [27]. We counted the number of habitat categories to which species were assigned in the Zip Code Zoo database (www.zipcodezoo.com) as an index of habitat niche breadth. To test whether habitat generalists showed the greatest improvement in sensitivity using the hybrid approach, we used linear regression to determine whether differences in sensitivity between the dynamic and hybrid approaches were positively associated with habitat niche breadth. In contrast, we expected the static approach to result in increased specificity relative to the dynamic approach (because the range expansion predicted using the dynamic approach may increase the number of misclassified absences). Therefore, we used linear regression to determine whether differences in specificity between the dynamic and static approaches were associated with proportions of absences in areas of range expansion. We reasoned that species for which the dynamic approach most overestimated range expansion may be dispersal limited, and unable to track changing climate [28]. Although we searched for information on known dispersal distances for species using online databases and literature searches, we were only able to obtain dispersal data for seven of our twelve study species. Because body size is positively correlated with dispersal distance for active dispersers [29], we used body size as a proxy for dispersal ability. We obtained data on maximum body mass from online databases (Animal Diversity Web, and Zip Code Zoo), which we log-transformed prior to analysis. We used linear regression to determine whether differences in specificity between the static and dynamic approaches were greatest for the species with the smallest body mass (i.e., the species expected to be most dispersal limited).

## Results

The PRISM data describe a warmer and slightly wetter climate across the contiguous United States in 1998–2002 compared with the 1967–1971 period. Annual precipitation in 1998–2002 averaged 768.5 mm compared with 763.3 mm in 1967–1971. Precipitation of the driest month was slightly greater in 1998–2002 (25.9 mm) than in 1967–1971 (25.4 mm), although precipitation of the wettest month was slightly lower (118.7 mm in 1998–2002 compared with 125.2 mm in 1967–1971). Temperature annual mean, maximum mean monthly temperature and minimum mean monthly temperature were all warmer in 1998–2002 compared with 1967–1971 (11.6°C vs 10.7°C, 30.8°C vs 30.3°C, −5.7°C vs −7.8°C, respectively), and the temperature annual range was lower in 1998–2002 (23.4°C) than in 1967–1971 (24.6°C).

Of the 12 species included in the study, previously published data suggest that eight experienced a northward range shift and four experienced a southward range shift (Table 1). In general, model sensitivity was greatest when presences were differentiated from absences using a prevalence criterion for the rarest species in the analysis (those represented by 262 or fewer occurrences in the calibration dataset, Table 1), whereas for more common species, model sensitivity was greatest when presence and absence was differentiated using the threshold that maximized kappa in the calibration dataset (Table 1). Although all 12 species have been suggested to have shifted their range in response to changing climate, static AUC values were higher than projected AUC values for at least one algorithm in nine out of 12 species (Table 1), suggesting that not all range shifts are consistent with a climate change model.

**Figure 3:**
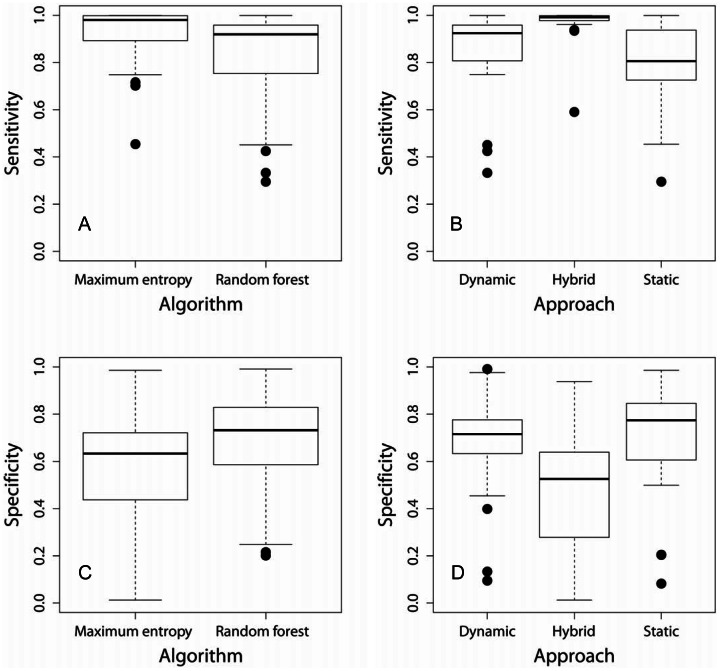
Box plots illustrating differences in climate envelope model sensitivity between models constructed with the maximum entropy and random forest algorithms (A), differences in sensitivity between models using three approaches that differ in the way they treat range expansion and contraction (B), differences in specificity between algorithms (C) and differences in specificity among approaches (D).

Average (dynamic) AUC values for the 12 random forest CEMs were 0.848±0.103 and for the maximum entropy CEMs average AUC was 0.868±0.097 (Table 1). The difference in AUC between algorithms was significant (χ^2^ = 5.021, df = 1, P = 0.025). Values of the true skill statistic averaged 0.524±0.196 for random forest CEMs and 0.551±0.258 for maximum entropy CEMs, but this difference was not statistically significant (χ^2^ = 0.324, df = 1, P = 0.569). Generalized linear mixed effects models describing effects of algorithm and approach on CEM sensitivity did not differ with or without interaction terms (χ^2^ = 0.865, df = 2, P = 0.649), so the significance of fixed effects was tested against the full model without interaction terms. Although maximum entropy models had greater sensitivity (0.92±0.122) than random forest models (0.82±0.202; Table 1, Figure 3A), the effect of algorithm on sensitivity was not significant (χ^2^ = 1.624, df = 1, P = 0.203). Mean sensitivity of the hybrid approach (0.97±0.082) was greater than either the dynamic (0.85±0.189) or static approach (0.80±0.183), but this difference was not statistically significant (χ^2^ = 3.902, df = 2, P = 0.142; Figure 3B).

Like the test for sensitivity, tests of all fixed effects on CEM specificity did not differ with or without interaction terms (χ^2^ = 1.132, df = 2, P = 0.568), so the significance of fixed effects was again tested against the full model without interaction terms. The effect of algorithm on specificity was significant (χ^2^ = 7.806, df = 1, P = 0.005), with random forest models having greater specificity (0.68±0.207) than maximum entropy models (0.56±0.263; Table 1, Figure 3C). The different approaches also varied in specificity (χ^2^ = 21.059, df = 2, P<0.001), with the hybrid approach having lower specificity (0.47±0.228) than either dynamic (0.67±0.219) or static approaches (0.71±0.218; Table 1, Figure 3D). Prediction maps for all species using the two algorithms and three approaches to model construction are included as supplementary figures ( Figures S1–S6).

**Figure 4:**
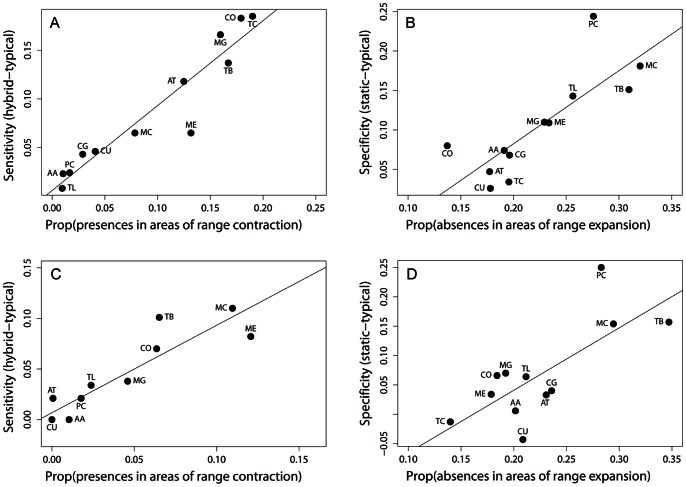
Relationships between differences in model sensitivity as a function of the proportion of presences in 1998–2002 that occurred in areas of range expansion (A and C for random forest and maximum entropy models, respectively) and differences in model specificity as a function of the proportion of absences in 1998–2002 that occurred in areas of range expansion (B and D for random forest and maximum entropy models, respectively).

**Figure 5:**
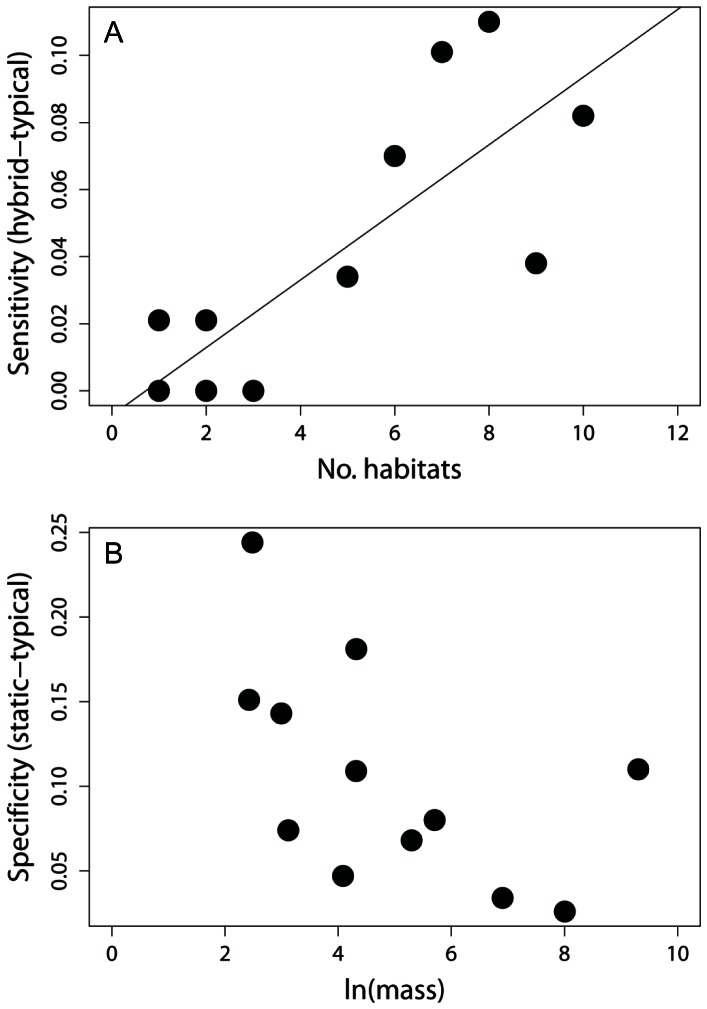
The improvement in sensitivity using a hybrid approach to climate envelope model evaluation that assumed no range contraction relative to a dynamic model in which ranges expanded and contracted in response to climate change was greatest for species that have been reported from relatively more habitat types (A), whereas differences in specificity between the dynamic approach and a static approach assuming no climate change were not significantly associated with body size (B).

Both random forest and maximum entropy models indicated that difference in sensitivity between dynamic and hybrid approaches increased with the proportion of presences occurring in areas of range contraction between *t_1_* and *t_2_* (F_1,10_ = 89.80, P<0.001 and F_1,10_ = 49.36, P<0.001; Figure 4A and 4C for random forest and maximum entropy models, respectively). The difference in specificity between dynamic and static approaches increased with the proportion of absences in areas of range expansion for both random forest and maximum entropy models (F_1,10_ = 18.24, P = 0.002 and F_1,10_ = 12.41, P = 0.006 for random forest and maximum entropy models, respectively; Figure 4B & 4D). For tests investigating the effect of niche breadth on changes in sensitivity between hybrid and dynamic approaches, we focused on results from maximum entropy models because sensitivity was greater, on average, than for random forest models (Table 1, Table 2). As hypothesized, species for which the hybrid approach yielded the greatest increase in sensitivity have been reported from more habitat types than species for which the hybrid approach had little effect on sensitivity (F_1,10_ = 17.98, P = 0.002; Figure 5A). We investigated whether small body size was associated with changes in specificity between static and dynamic approaches for random forest models, because specificity was greater than for maximum entropy models (Table 1, Table 2). There was suggestive, but not significant relationship indicating that differences in specificity between static and dynamic approaches were greatest for the smallest-bodied species (F_1,10_ = 4.30, P = 0.065; Figure 5B).

**Table 2:** Body size and habitat niche breadth for 12 species of resident North American breeding birds.

Common Name	Number of land cover types used	ln (mass)
Wild turkey	9	9.31
Greater sage grouse	1	8.01
Greater prairie chicken	2	6.91
Gambel’s quail	3	5.30
Red-headed woodpecker	10	4.32
Red-bellied woodpecker	8	4.32
Fish crow	6	5.71
Carolina chickadee	1	2.49
Carolina wren	5	2.99
Bewick’s wren	7	2.43
Bachman’s sparrow	2	3.12
Tricolored blackbird	2	4.09

Trait data were obtained from online natural history databases.

## Discussion

The choice of modeling algorithm, rather than the approach to model evaluation, had the greatest overall effect on sensitivity, whereas specificity was affected by both algorithm and approach. The hybrid approach to CEM evaluation did not result in a significant overall increase in model sensitivity, but did result in decreased specificity relative to dynamic and static approaches (Figure 3B & 3D). These general trends, however, obscure substantial species-specific responses that illustrate how users can create models that vary in their assumptions about climate change effects on range expansion and contraction depending on characteristics of the species in question and the relative importance of omission and commission error. For example, we were able to correctly almost 30% more presences for some species using the hybrid approach compared with the dynamic approach (Table 1), although at a cost of reduced specificity. Improved sensitivity was the result of an increase in presences that were correctly classified using the hybrid approach, but misclassified using the dynamic approach because they occurred in areas of range contraction between *t_1_* and *t_2_* (Figure 4A & 4C). Species that experienced the greatest improvement in sensitivity using the hybrid approach were reported from more land cover types than species for which dynamic and hybrid approaches differed little in sensitivity. Overall, specificity was similar using both the dynamic and static approaches, although specificity tended to be greatest using the static approach for species experiencing the greatest number of absences in areas of range expansion. However, for three species evaluated at least one algorithm indicated that sensitivity of the static approach was at least as good as with a dynamic approach, and for nine species the static AUC was greater than the dynamic AUC, suggesting that climate change may not necessarily always underlie purported climate-induced range shifts.

Although differences in model sensitivity between approaches were not statistically significant overall, our use of alternative approaches to CEM evaluation provides a useful framework for evaluating alternative explanations about climate change effects on species. Comparing CEMs that make competing predictions about climate-induced range expansion and contraction may have important implications for natural resource management decisions made on the basis of CEMs. If managing for an endangered or invasive species, for example, for which the priority is to identify all possible suitable areas (e.g., a prioritization of reduced omission error at the expense of increased commission error), a hybrid model that includes the possibility of range expansion but not contraction may be preferred. On the other hand, increased specificity (which may be desirable, for example, when identifying areas unlikely to be suitable for a problematic invasive species) may be achieved using the random forest algorithm (an observation consistent with the random forest’s tendency to reduce overprediction, [20]).

The dynamic approach to climate envelope modeling tended to overestimate the extent of range contraction that species experienced (Figure 3A & 3C). We found that species for which the dynamic CEM approach most overestimated range contractions have been reported from many different land cover types. It has been suggested that habitat generalists are buffered from the negative effects of habitat fragmentation [27], and our work suggests that generalists may also be buffered to some degree from climate-induced range contractions. Alternatively, it has been suggested that generalist species may be better able to track changing climate than habitat specialists [30]. Given the potential for complex interactions between species traits and changing climate, we suggest that more work relating species traits to the extinction debt [31] accumulated as a result of climate change is needed.

Although specificity showed little overall difference between dynamic and static approaches, there were differences between the two approaches for individual species, and a tendency for the static approach to perform best when the dynamic approach overestimated the number of absences in areas of range expansion. We found a marginally non-significant relationship suggesting that smaller-bodied species experienced that greatest improvement in specificity using the static CEM approach. Although smaller body size generally equates to dispersal limitation [29], there is substantial error in this generalization. Unfortunately, dispersal distances are unreported for many of the species reported here so we are unable to test for direct effects of dispersal limitation on differences in specificity. Although transplant experiments in which species successfully establish themselves in areas of climate suitability attest to the potential importance of dispersal limitation as a factor that prevents species from tracking changing climate [30], little work is available to suggest that the most dispersal limited species are least able to track climate change. Interpreting model specificity is also complicated by the uncertain nature of absences [32], because species are likely present but unobserved at some locations categorized as absences. However, the suggestive relationship between body size and improved specificity using a static approach may indicate that small bodied species are not able to track changing climate as efficiently as larger-bodied species with greater dispersal abilities.

Although climate may be an important determinant of species distributions at broad spatial scales [4], it is not necessarily the most important factor in circumscribing species’ geographic ranges. For many species, habitat loss [33] or other factors (e.g., competition and dispersal, [34]) may be as or more important than climate in determining current or future geographic distributions. Some of the species included in our analysis have experienced range expansion at least partly because of factors other than climate change (e.g., the Wild turkey has been the target of reintroduction efforts in parts of its range, [35]). Furthermore, for three species in our analysis, at least one algorithm showed that a static approach assuming no climate change classified at least as many *t_2_* presences as the dynamic approach assuming climate change effects, and for one species (the Tricolored blackbird), the static approach had greater sensitivity using both algorithms (Table 1). Indeed, the relative improvement in sensitivity between dynamic and static approaches may be a useful metric of the magnitude of climate change effects on species. It may also be expected that performance of some models would be improved by adding data describing non-climate environmental conditions (e.g., land cover) in addition to climate data. For example, the Fish crow, *Corvus ossifragus*, is associated with wetland habitats in the eastern United States, suggesting that models that do not include the distribution of wetland habitat may underperform relative to models that include habitat. Consistent with this expectation, experimental niche models for the fish crow that included land cover data had greater sensitivity than the models we report on here (J. I. Watling, unpublished). We also acknowledge that non-climate driven spatial variation in population dynamics (i.e., metapopulation structure) may play a role in driving the range shifts we describe here.

Our results suggest cautious optimism when using predictions from CEMs to infer climate change effects on species, and we demonstrate how model construction may be manipulated to best suit alternative model needs. We suggest that the use of alternative CEMs that make different assumptions about range expansion and contraction can help inform an understanding of climate change effects on species. However, our results also suggest that CEMs do not unambiguously implicate climate change as a driver of observed species range shifts in many cases, underscoring the importance of considering additional factors when considering species range shifts through time.

## Supporting Information

Figure S1 **Figure panels with binary prediction maps indicating areas of suitable (brick red) and unsuitable (dark yellow) climate for 12 species of resident North American breeding birds.** Models were calibrated on climate conditions for the 1967–1971 period and projected using climate conditions for 1998–2002. Presences (dark circles) and absences (white circles) from 1998–2002 surveys are indicated. Illustrated are predictions from a random forest model using the dynamic approach described in the text.
(TIF)

Figure S2 **Figure panels with binary prediction maps indicating areas of suitable (brick red) and unsuitable (dark yellow) climate for 12 species of resident North American breeding birds.** Models were calibrated on climate conditions for the 1967–1971 period and projected using climate conditions for 1998–2002. Presences (dark circles) and absences (white circles) from 1998–2002 surveys are indicated. Illustrated are predictions from a maximum entropy model using the dynamic approach described in the text.
(TIF)

Figure S3 **Figure panels with binary prediction maps indicating areas of suitable (brick red) and unsuitable (dark yellow) climate for 12 species of resident North American breeding birds.** Models were calibrated on climate conditions for the 1967–1971 period and projected using climate conditions for 1998–2002. Presences (dark circles) and absences (white circles) from 1998–2002 surveys are indicated. Illustrated are predictions from a random forest model using the static approach described in the text.
(TIF)

Figure S4 **Figure panels with binary prediction maps indicating areas of suitable (brick red) and unsuitable (dark yellow) climate for 12 species of resident North American breeding birds.** Models were calibrated on climate conditions for the 1967–1971 period and projected using climate conditions for 1998–2002. Presences (dark circles) and absences (white circles) from 1998–2002 surveys are indicated. Illustrated are predictions from a maximum entropy model using the static approach described in the text.
(TIF)

Figure S5 **Figure panels with binary prediction maps indicating areas of suitable (brick red) and unsuitable (dark yellow) climate for 12 species of resident North American breeding birds.** Models were calibrated on climate conditions for the 1967–1971 period and projected using climate conditions for 1998–2002. Presences (dark circles) and absences (white circles) from 1998–2002 surveys are indicated. Illustrated are predictions from a random forest model using the hybrid approach described in the text.
(TIF)

Figure S6 **Figure panels with binary prediction maps indicating areas of suitable (brick red) and unsuitable (dark yellow) climate for 12 species of resident North American breeding birds.** Models were calibrated on climate conditions for the 1967–1971 period and projected using climate conditions for 1998–2002. Presences (dark circles) and absences (white circles) from 1998–2002 surveys are indicated. Illustrated are predictions from a maximum entropy model using the hybrid approach described in the text.
(TIF)
