# Supplementary figures and images for: Validating Predictions from Climate Envelope Models
Source: PLoS One. 2013 May 23;8(5):e63600. doi: 10.1371/journal.pone.0063600 (PMC3662712; doi:10.1371/journal.pone.0063600)

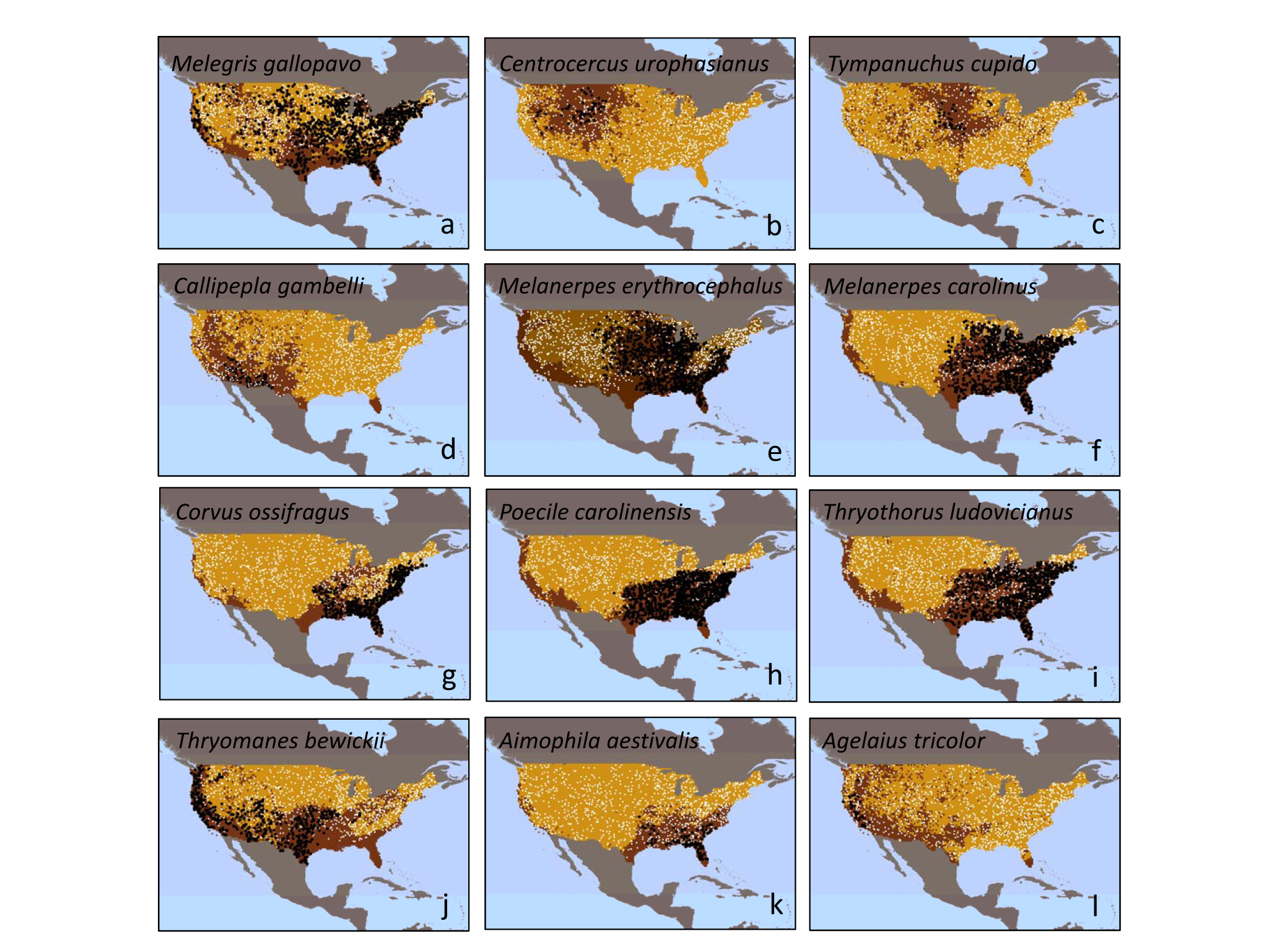

Supplement: Figure S1 — Figure panels with binary prediction maps indicating areas of suitable (brick red) and unsuitable (dark yellow) climate for 12 species of resident North American breeding birds. Models were calibrated on climate conditions for the 1967–1971 period and projected using climate conditions for 1998–2002. Presences (dark circles) and absences (white circles) from 1998–2002 surveys are indicated. Illustrated are predictions from a random forest model using the dynamic approach described in the text. (TIF) [file pone.0063600.s001.tif]

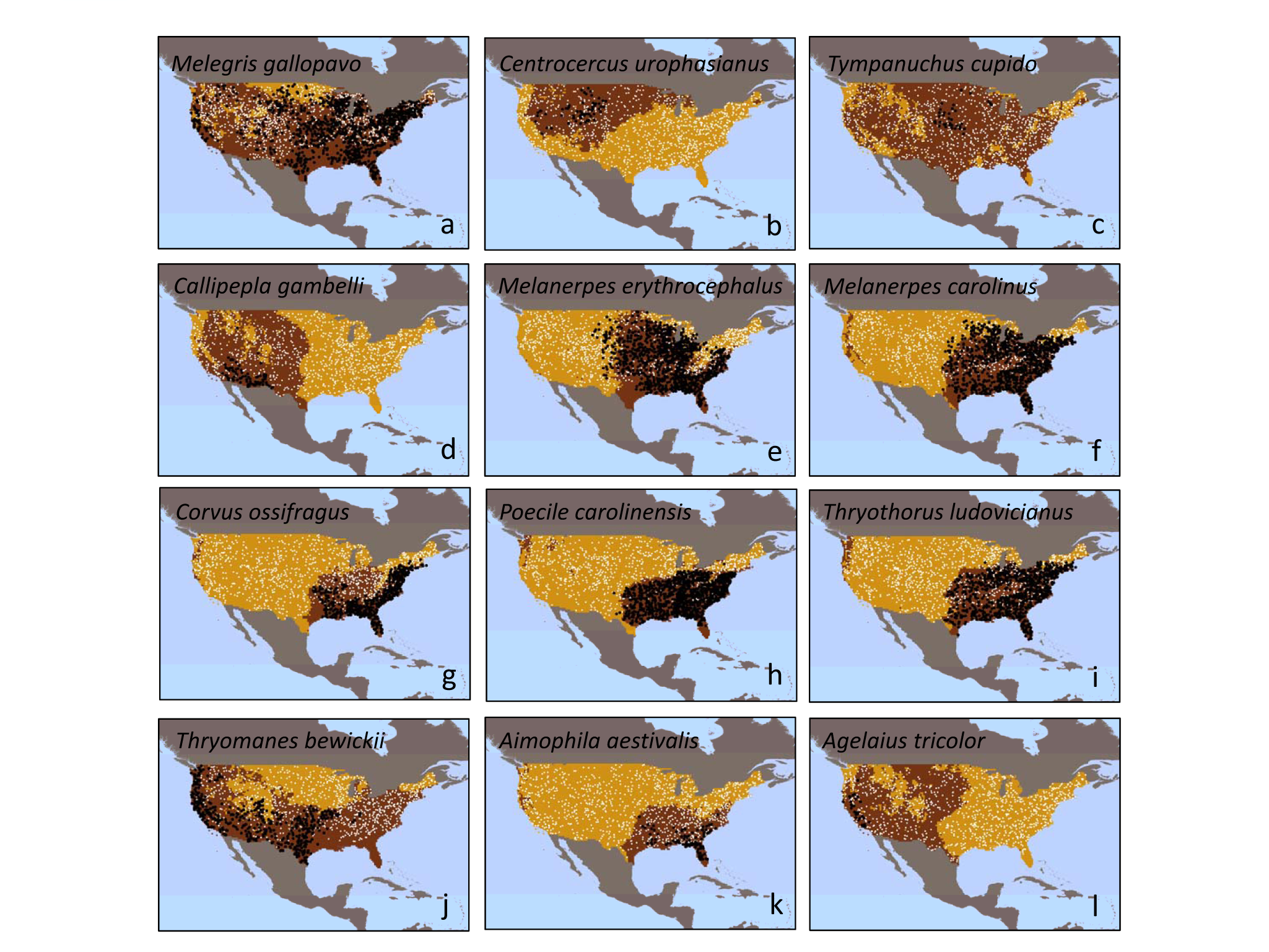

Supplement: Figure S2 — Figure panels with binary prediction maps indicating areas of suitable (brick red) and unsuitable (dark yellow) climate for 12 species of resident North American breeding birds. Models were calibrated on climate conditions for the 1967–1971 period and projected using climate conditions for 1998–2002. Presences (dark circles) and absences (white circles) from 1998–2002 surveys are indicated. Illustrated are predictions from a maximum entropy model using the dynamic approach described in the text. (TIF) [file pone.0063600.s002.tif]

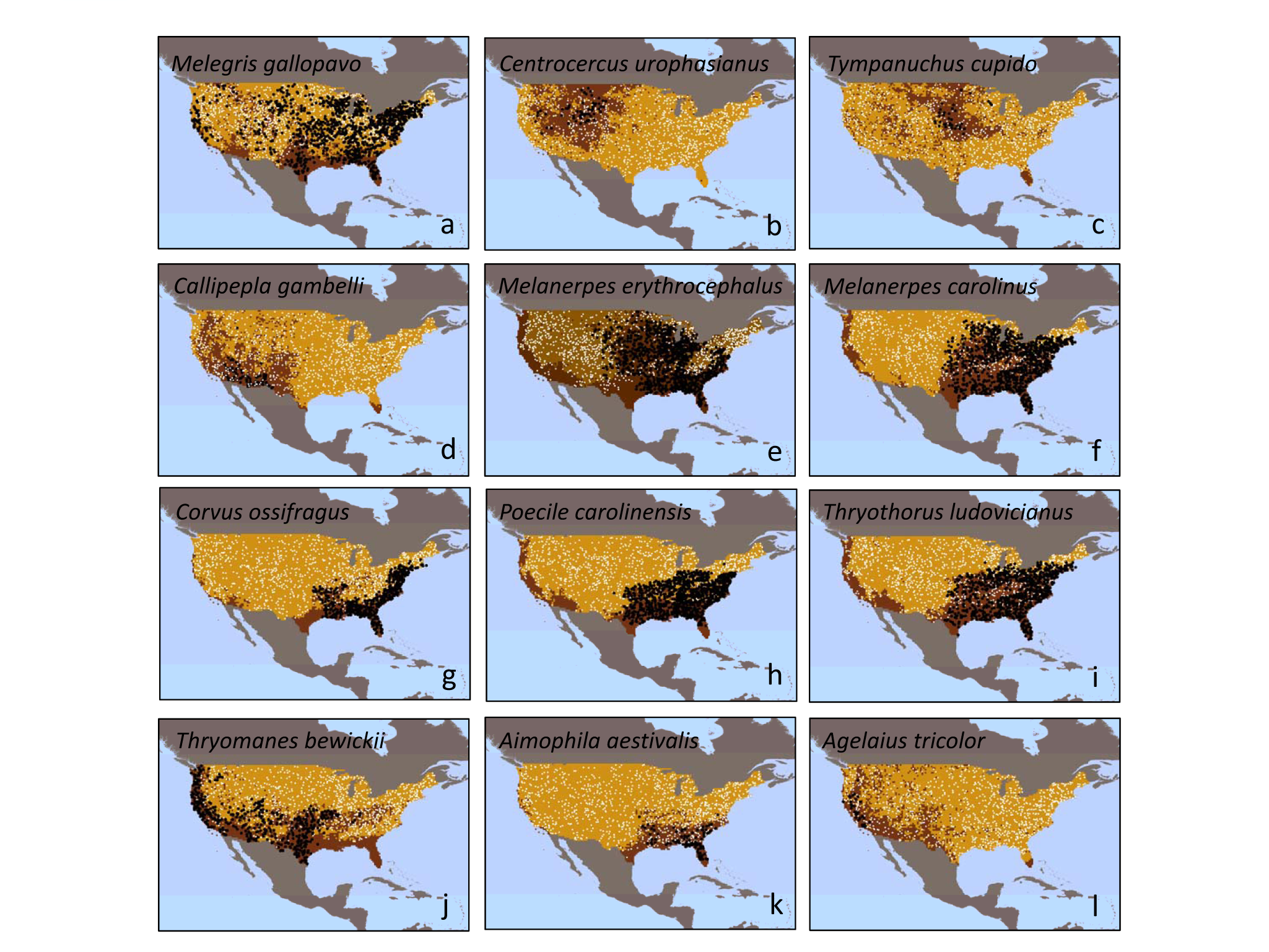

Supplement: Figure S3 — Figure panels with binary prediction maps indicating areas of suitable (brick red) and unsuitable (dark yellow) climate for 12 species of resident North American breeding birds. Models were calibrated on climate conditions for the 1967–1971 period and projected using climate conditions for 1998–2002. Presences (dark circles) and absences (white circles) from 1998–2002 surveys are indicated. Illustrated are predictions from a random forest model using the static approach described in the text. (TIF) [file pone.0063600.s003.tif]

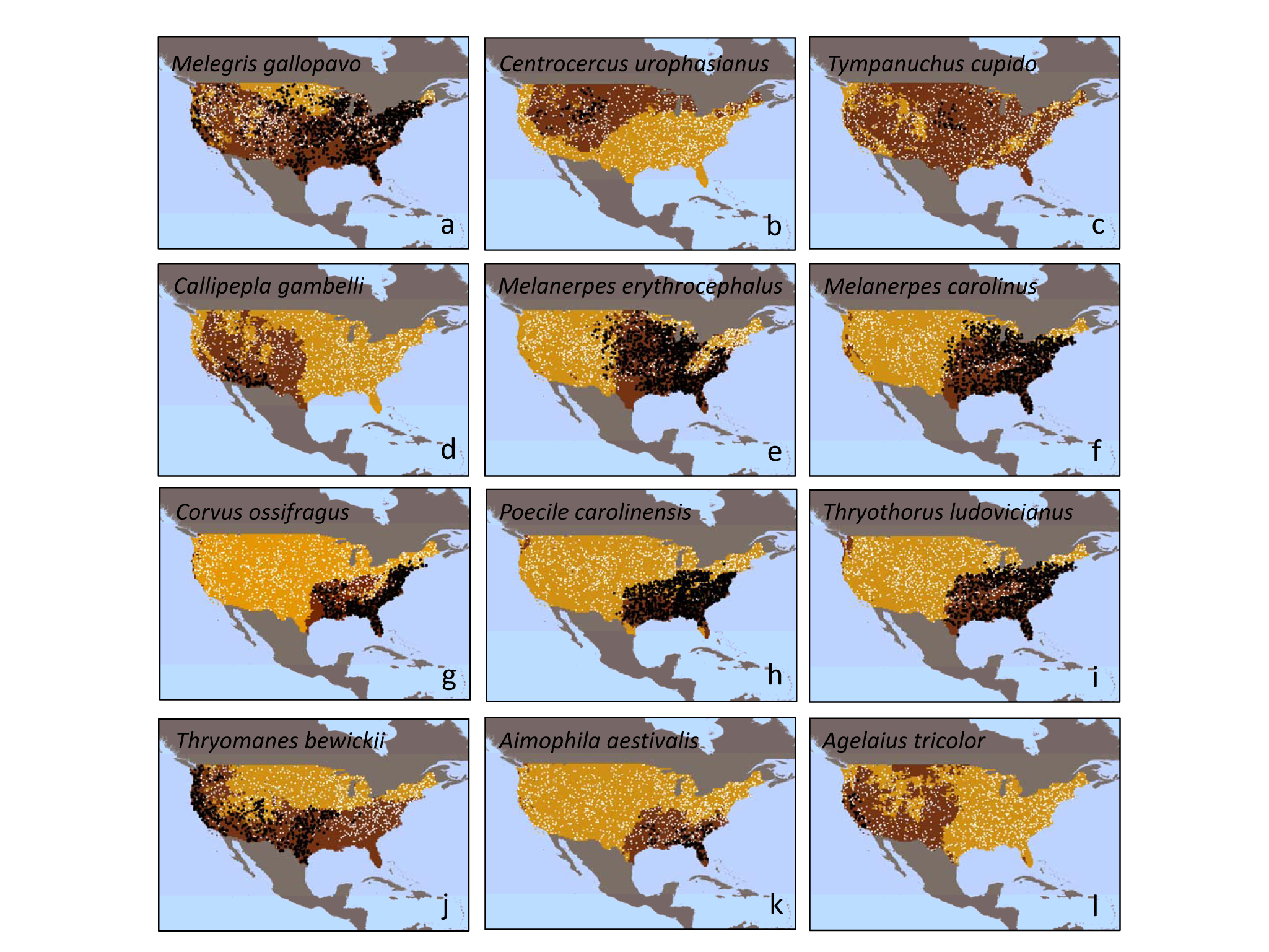

Supplement: Figure S4 — Figure panels with binary prediction maps indicating areas of suitable (brick red) and unsuitable (dark yellow) climate for 12 species of resident North American breeding birds. Models were calibrated on climate conditions for the 1967–1971 period and projected using climate conditions for 1998–2002. Presences (dark circles) and absences (white circles) from 1998–2002 surveys are indicated. Illustrated are predictions from a maximum entropy model using the static approach described in the text. (TIF) [file pone.0063600.s004.tif]

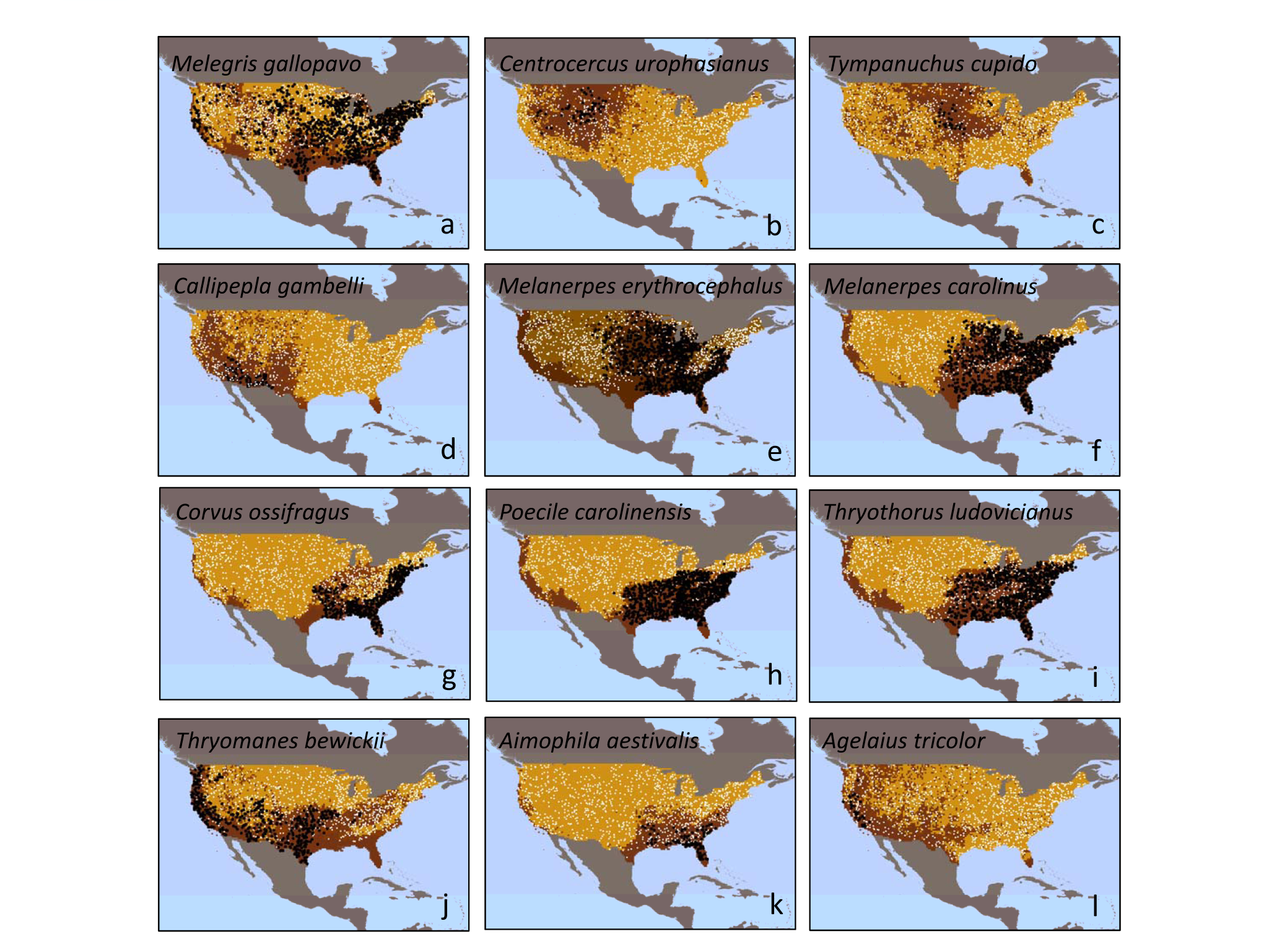

Supplement: Figure S5 — Figure panels with binary prediction maps indicating areas of suitable (brick red) and unsuitable (dark yellow) climate for 12 species of resident North American breeding birds. Models were calibrated on climate conditions for the 1967–1971 period and projected using climate conditions for 1998–2002. Presences (dark circles) and absences (white circles) from 1998–2002 surveys are indicated. Illustrated are predictions from a random forest model using the hybrid approach described in the text. (TIF) [file pone.0063600.s005.tif]

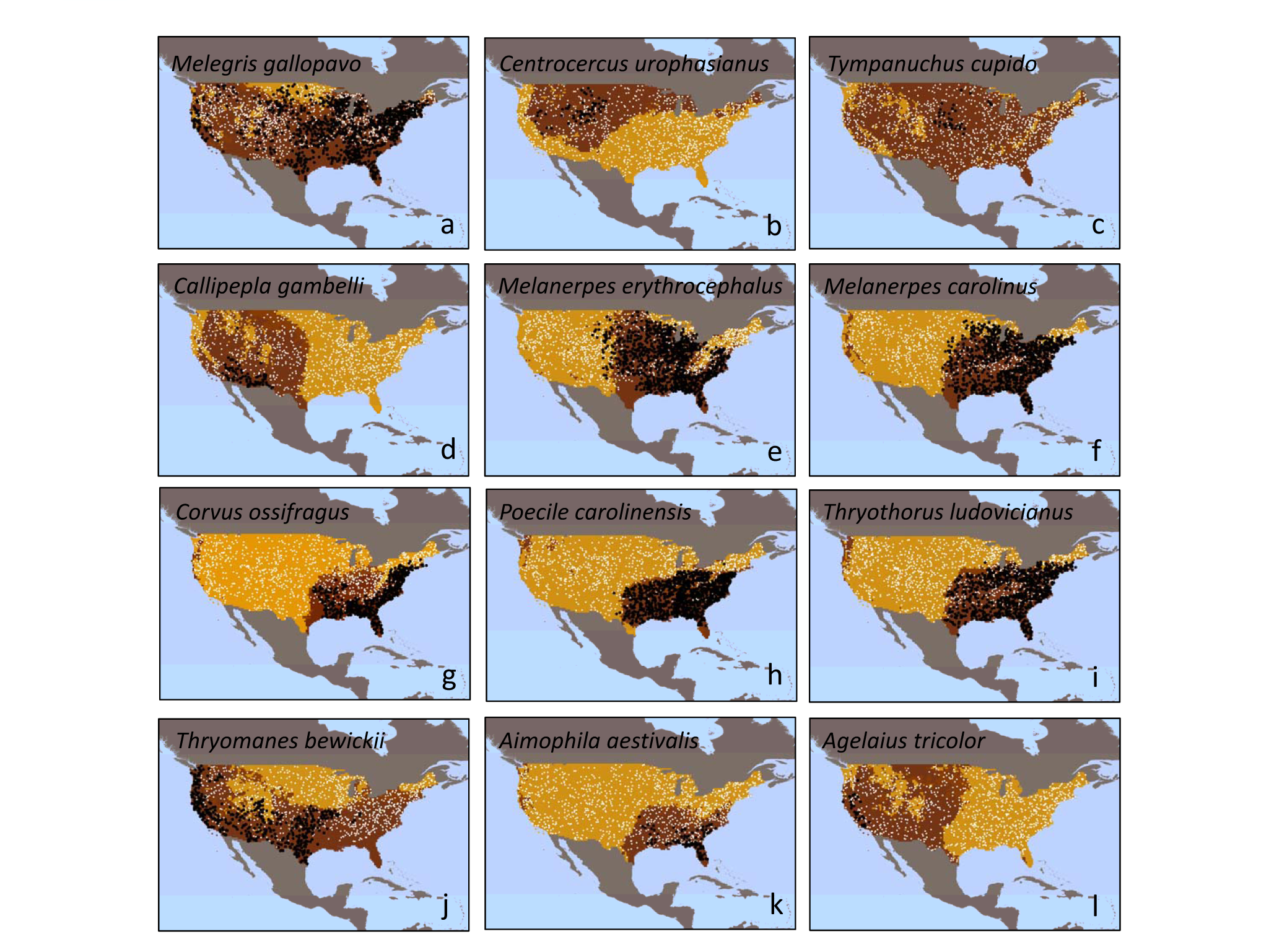

Supplement: Figure S6 — Figure panels with binary prediction maps indicating areas of suitable (brick red) and unsuitable (dark yellow) climate for 12 species of resident North American breeding birds. Models were calibrated on climate conditions for the 1967–1971 period and projected using climate conditions for 1998–2002. Presences (dark circles) and absences (white circles) from 1998–2002 surveys are indicated. Illustrated are predictions from a maximum entropy model using the hybrid approach described in the text. (TIF) [file pone.0063600.s006.tif]
